# Effects of brief exposure to misinformation about e-cigarette harms on Twitter on knowledge and perceptions of e-cigarettes

**DOI:** 10.1177/20552076221116780

**Published:** 2022-08-02

**Authors:** Jessica Liu, Caroline Wright, Olga Elizarova, Jennifer Dahne, Jiang Bian, Philippa Williams, Brittany Zulkiewicz, Andy SL Tan

**Affiliations:** 1 Department of Social and Behavioral Sciences, 1857 Harvard T. H. Chan School of Public Health, Boston, MA, USA; 2 Population Health Sciences, 152331 Bristol Medical School, University of Bristol, Bristol, UK; 3 Play Collaborate Change, Boston, MA, USA; 4 Department of Psychiatry and Behavioral Sciences, College of Medicine, 2345 Medical University of South Carolina, Charleston, SC, USA; 5 Hollings Cancer Centre, 2345 Medical University of South Carolina, Charleston, SC, USA; 6 Health Outcomes & Biomedical Informatics, 12233 College of Medicine, University of Florida, Gainesville, FL, USA; 7 310547 Annenberg School for Communication, University of Pennsylvania, Philadelphia, PA, USA

**Keywords:** public health, disease, health communications, general, smoking, lifestyle, twitter, media, online

## Abstract

**Background:**

This study examined whether exposure to misinformation found on Twitter about e-cigarette harms leads to inaccurate knowledge and misperceptions of harms of e-cigarette use among cigarette smokers.

**Methods:**

We conducted an online randomized controlled experiment in November 2019 among an online sample of 2400 adult US and UK cigarette smokers who did not currently use e-cigarettes. Participants viewed four tweets in one of four conditions: 1) e-cigarettes are as or more harmful than smoking, 2) e-cigarettes are completely harmless, 3) e-cigarette harms are uncertain and 4) control (physical activity). Outcomes were knowledge about e-cigarettes and harm perceptions of e-cigarette use for five diseases. We conducted multiple logistic and linear regressions to analyze the effect of experimental conditions on outcomes, controlling for baseline knowledge and perceived harms.

**Findings:**

Participants in the ‘as or more harmful’ condition (vs. control group) had higher odds of accurate knowledge about e-cigarettes containing toxic chemicals (*p* < 0.001), not containing only water vapor (p < 0.001) and containing formaldehyde (*p* < 0.001). However, these participants had lower odds of accurate knowledge that e-cigarettes did not contain tar (*p* < 0.001) and contained fewer toxins than cigarettes (*p* < 0.001). Exposure to ‘as or more harmful’ tweets also increased harm perceptions for five diseases (all *p* < 0.001), with the greatest effect observed for lung cancer (β = 0.313, *p* < 0.001). This effect was greater among UK participants for all diseases.

**Interpretation:**

Brief exposure to misinformation on Twitter reduced accurate knowledge of the presence of tar and the level of toxins compared with smoking and increased harm perceptions of e-cigarette use.

## Introduction

E-cigarettes are a contentious public health issue in both the US and UK.^1^ Current evidence indicates that the short-term harm caused by e-cigarettes equates to only 5% of the harm caused by regular tobacco cigarettes.^2^ However, limited evidence on the long-term health effects of e-cigarettes has led to debate over the risk trade-offs associated with their use.^3^ The prevalence of current e-cigarette use, defined as use in the past 30 days, has remained stable in recent years and is currently estimated to be between 5.5% and 6.3% amongst UK adults in 2020,^4^ and 4.5% among US adults in 2019.^5^ Uptake is greatest amongst current and former smokers, and the most commonly cited reason for their use is to aid smoking cessation.^2^ Consequently, e-cigarettes may help to reduce smoking-related morbidity and mortality by providing a viable alternative for adult smokers.^6^

Despite current evidence, misperceptions of the relative harms of e-cigarettes are increasing.^7^ The proportion of UK smokers who perceived e-cigarettes to be just as harmful as combustible cigarettes increased from 26% in 2014 to 38% in 2020.^4^ Similarly, harm perceptions among US smokers increased from 36.4% in 2017 to 43.0% in 2018.^8^ These findings are significant for public health interventions, as evidence has shown that for every 1% increase in misperceptions among current smokers, mean prevalence of e-cigarette use decreases by 0.48%.^9^ There is a need in the field to prioritize understanding and measuring how cigarette smokers perceive e-cigarettes and to understand why they continue to use combustible cigarettes, especially as newer forms of e-cigarettes are introduced in the marketplace. A greater understanding of the effects of harm perceptions of e-cigarettes may help to inform public health strategies to identify and counteract misinformation.^10^

One potential cause of these misperceptions may be exposure to misinformation on social media.^11^ Misinformation can be defined as information that is incorrect or misleading, and misperceptions caused by exposure to misinformation are difficult to correct once established.^11^ Evidence shows that misinformation spreads more quickly than accurate information online and can discourage positive health behaviors.^12^ Thus, it is important to understand how exposure to social media and other messaging can influence knowledge around e-cigarettes.

Based on the general consensus in the evidence base that e-cigarettes are relatively less harmful than regular cigarettes,^2^ we designed a randomized controlled experiment to briefly expose participants to two types of misinformation about e-cigarettes: ‘e-cigarettes are just as or more harmful than smoking’ and ‘e-cigarettes are completely harmless.’ A third ‘uncertainty’ condition was also included to reflect current uncertainties amongst the public and in the evidence base, as research indicates that exposure to scientific uncertainty can influence e-cigarette perceptions.^13^

Prior analyses from this study have found that after brief exposure to tweets e-cigarettes were just as or more harmful than smoking decreased intentions to use e-cigarettes.^14^ Participants were also more likely to engage with (e.g. share, like) tweets that e-cigarettes were just as or more harmful than smoking,^15^ we also found that affective responses and perceived relative harm following exposure to misinformation about e-cigarette harms may mediate the relationship with intention to purchase e-cigarettes.^16^

This current study examined the effect of brief exposure to misinformation found on Twitter about e-cigarette harms on smoker's knowledge and harm perceptions of e-cigarettes relative to combustible cigarettes, across US versus UK populations. We define knowledge as being aware of factual information about e-cigarette constituents and health effects of e-cigarette use. Harm perceptions are defined as beliefs about the likelihood of developing various health problems when switching completely from smoking to using e-cigarettes. We hypothesize that exposure to misinformation that e-cigarettes are just as or more harmful than smoking will be associated with increased knowledge of harms and increased harm perceptions of e-cigarettes among adult smokers.

## Methods

### Study design

This study used a randomized controlled experimental design conducted online through the Prodege consumer research panel in the US and UK.^14^ Ethics approval was obtained by the University of Bristol's Institutional Review Board.

### Participants

Participants were 2400 self-reported adult smokers aged 18 years and older who did not currently use e-cigarettes and lived in the US or UK. An equal number of US and UK participants were recruited in November 2019. Participants were members of the Prodege consumer research panel and were recruited through email invitations, telephone alerts, banners and messaging on websites and online communities. Participants provided informed consent electronically through the survey platform and received reward points for taking part as per Prodege policies.

### Randomization and masking

Following eligibility screening, providing informed consent, and completing baseline measures, participants were then randomized to one of four experimental conditions: 1) E-cigarettes are as or more harmful than smoking; 2) E-cigarettes are completely harmless; 3) Uncertainty about e-cigarette harms; and 4) Control (physical activity) in a 1:1:1:1 ratio using the in-built least-fill randomizer function on the Prodege survey platform. These ensured participant characteristics were distributed evenly across message conditions, preventing selection bias and minimizing the risk of confounding factors.

### Message stimuli selection

A random sample of 1% (*n* = 449) of tweets was selected and reviewed by the team to determine their suitability as a message stimulus for the experimental conditions. Further details of the message stimuli selection from Twitter and the machine learning algorithm used are described elsewhere.^14^

### Procedures

**Figure 1.**
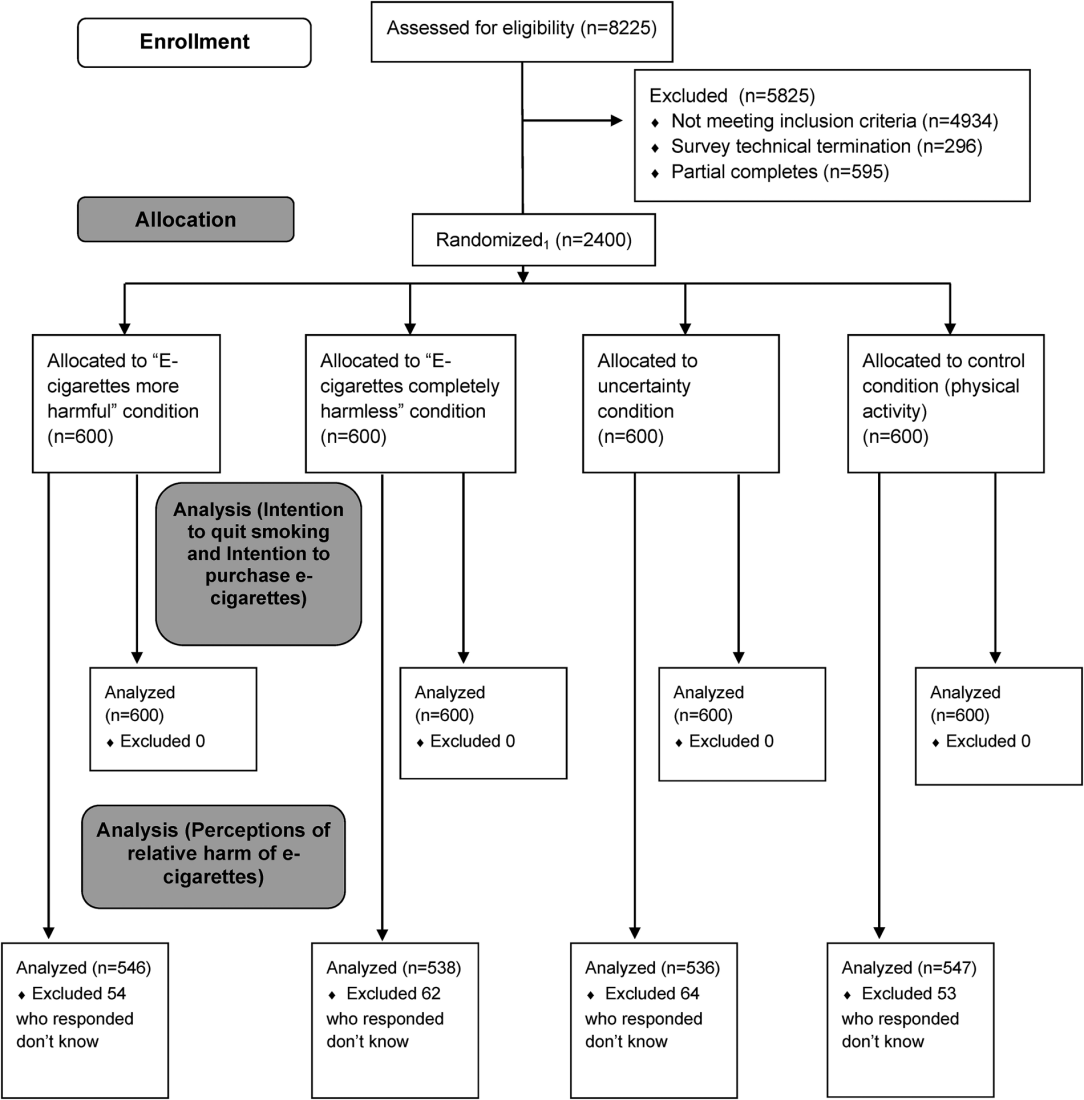
CONSORT flow diagram. 1. Survey recruitment used a least-fill approach; as a respondent came in, they were assigned to the exposure with the lowest complete count.

After randomization, participants viewed four consecutive tweets in random order and were asked brief questions on their perceived effectiveness, likelihood of engaging (e.g. like, share), and emotions produced by the message (results presented elsewhere).^15,16^ After exposure, participants completed post-test measures of knowledge and harm perceptions. They then answered questions on smoking behavior, prior e-cigarette misinformation exposure, social media use and demographic characteristics. Participants were debriefed with accurate information about e-cigarette harms and given information about local smoking cessation services (see Figure 1: CONSORT diagram).

### Outcomes

*Knowledge of e-cigarettes.* Knowledge measures, asked at both baseline and post-exposure, were based on previous research.^17–21^ Participants were asked to respond ‘True’, ‘False’ or ‘Don't know’ to six statements about e-cigarettes (the correct answers are in parentheses):

1. ‘E-cigarettes do not contain any of the toxic chemicals that can be found in regular cigarettes’ (false)
2. ‘Vapor from e-cigarettes contains only water vapor’ (false)
3. ‘Vapor from e-cigarettes contains formaldehyde’ (true)
4. ‘E-cigarettes contain tar, which can cause lung cancer’ (false)
5. ‘Smokers who switch to e-cigarettes may breathe fewer toxins’ (true)
6. ‘E-cigarette use or vaping leads to an incurable condition known as “popcorn lung”’ (false)

Each statement will be referred to using the following key terms: Toxic Chemicals, Water Vapor, Formaldehyde, Tar, Fewer Toxins, and Popcorn Lung. Participant responses were recoded into a binary outcome of knowledge about each statement (correct answers = 1, incorrect answer or ‘don't know’ = 0).

*Harm perceptions of switching completely to e-cigarettes.* Measures of harm perceptions, asked at both baseline and post-exposure, were based on previous research.^22^ To measure the perceived likelihood of developing health problems, participants were asked: “Imagine that you stopped smoking regular cigarettes and only used e-cigarettes/vapes. How likely do you think it is that using e-cigarettes/vapes regularly would cause you to develop each of the following diseases in the next 10 years?” (Lung cancer, Heart disease, Mouth or throat cancer, Chronic obstructive pulmonary disease (COPD), and Stroke). Participants rated perceived likelihood on a 5-point Likert scale ranging from “extremely unlikely” (1) to “extremely likely” (5).

### Statistical analysis

Effect size estimates in the outcome variables as a function of message condition were conducted previously by the research team using GPower ver.3.1.^23^ This gave a final sample size of 2,400, with 600 in each arm, to ensure sufficient power to detect small effects between conditions among adult smokers (f = 0.07). Univariate analyses were performed for all study variables stratified by country. Logistic regression analyses were conducted to predict correct post-exposure knowledge of each statement by experimental condition, controlling for baseline knowledge. Linear regression analyses were conducted to predict post-exposure harm perceptions of each of the five diseases by experimental condition, controlling for baseline measures of harm perceptions. Further regression analyses stratified by country compared the effect of exposure on study outcomes for US and UK participants. For all regression analyses, the control condition was the referent category. There were no missing values for our outcomes of interest Sensitivity analysis including country as a covariate was utilized owing to the differences in baseline measurements between US and UK participants. All analyses were conducted using R.^24^

## Results

### Participants

Participants were 2400 current adult smokers, aged 18–84 years (mean = 47.0, SD = 14.6), and 46.8% were female. Participant characteristics were evenly distributed across experimental conditions (Table 1). Further details are reported in the main outcomes paper.^14^

**Table 1.** Characteristics of study sample by country and condition.

	US				UK			
Characteristics	Condition 1 (*n* = 300)	Condition 2 (*n* = 300)	Condition 3 (*n* = 300)	Condition 4 (*n* = 300)	Condition 1 (*n* = 300)	Condition 2 (*n* = 300)	Condition 3 (*n* = 300)	Condition 4 (*n* = 300)
Age: Mean (SD)								
	50.5 (13.6)	50.0 (13.6)	50.0 (14.7)	50.3 (13.5)	44.1 (14.6)	44.2 (14.4)	44.0 (14.8)	42.8 (14.6)
Sex: No. (%)								
Female	153 (51.0)	154 (51.3)	154 (51.3)	140 (46.7)	126 (42.0)	136 (45.3)	125 (41.7)	135 (45.0)
Gender: No. (%)								
Man	148 (49.3)	148 (49.3)	146 (48.7)	160 (53.3)	172 (57.3)	163 (54.3)	175 (58.3)	164 (54.7)
Woman	150 (50.0)	152 (50.7)	154 (51.3)	139 (46.3)	124 (41.3)	136 (45.3)	123 (41.0)	135 (45.0)
Transgender	0 (0.0)	0 (0.0)	0 (0.0)	1 (0.3)	3 (1.0)	0 (0.0)	1 (0.3)	0 (0.0)
Nonbinary/Genderqueer	2 (0.7)	0 (0.0)	0 (0.0)	0 (0.0)	1 (0.3)	1 (0.3)	1 (0.3)	1 (0.3)
US Race: No. (%)								
White	206 (68.7)	214 (71.3)	211 (70.3)	220 (73.3)				
Black or African American	51 (17.0)	47 (15.7)	52 (17.3)	51 (17.0)				
Other ethnicity	43 (14.3)	39 (13.0)	37 (12.3)	29 (9.7)				
UK Ethnicity: No. (%)								
White					284 (94.7)	276 (92.0)	278 (92.7)	282 (94.0)
Other ethnicity					16 (5.3)	24 (8.0)	22 (7.3)	18 (6.0)
Education								
High/ Secondary school or below	83 (27.7)	99 (33.0)	91 (30.3)	89 (29.7)	118 (39.3)	126 (42.0)	122 (40.7)	129 (40.3)
Some college/further education college	111 (37.0)	122 (40.7)	123 (41.0)	110 (36.7)	110 (36.7)	103 (34.3)	105 (35.0)	105 (35.0)
College/University degree or higher	106 (35.3)	79 (26.3)	86 (28.7)	101 (33.7)	72 (24.0)	71 (23.7)	73 (24.3)	66 (22.0)
Smoking status: Mean (SD)								
No. days smoked in last 30 days	28.9 (4.2)	27.8 (5.9)	27.7 (5.9)	28.1 (5.4)	27.5 (6.3)	27.4 (6.9)	26.7 (7.7)	27.1 (7.0)
E-cigarette use: No. (%)								
Ever used e-cigarettes	155 (51.7)	156 (52.0)	148 (49.3)	142 (47.3)	162 (54.0)	176 (58.7)	148 (49.3)	152 (50.7)
FTND* category: No. (%)								
Low	71 (23.7)	66 (22.0)	85 (28.3)	82 (27.3)	92 (30.7)	87 (29.0)	99 (33.0)	87 (29.0)
Low to moderate	85 (28.6)	87 (29.0)	85 (28.3)	78 (26.0)	85 (28.3)	84 (28.0)	76 (25.3)	69 (23.0)
Moderate	119 (39.7)	126 (41.0)	105 (35.0)	107 (35.7)	99 (33.0)	104 (34.7)	106 (35.3)	121 (40.3)
Higher	25 (8.0)	21 (7.0)	25 (8.33)	33 (11.0)	24 (8.0)	25 (8.33)	19 (6.33)	23 (7.67)
Ever looked for e-cigarette information, No. (%)								
Yes	75 (25.0)	81 (27.0)	58 (19.3)	76 (25.3)	72 (24.0)	78 (26.0)	74 (24.7)	82 (27.3)
Social Media use: Mean (SD)								
Range 0–8	1.62 (1.46)	1.76 (1.49)	1.80 (1.63)	1.72 (1.56)	2.11 (1.65)	2.24 (1.71)	2.19 (1.77)	2.19 (1.64)
Daily Internet use: Mean (SD)								
Range 0–24	6.59 (4.77)	6.89 (4.72)	6.24 (4.37)	6.48 (4.37)	5.18 (3.70)	5.54 (4.01)	5.80 (3.80)	5.65 (3.92)

### Knowledge of e-cigarette harms

At baseline, the most frequent score of correct answers was two out of six (28.92%, *n* = 694), and only two participants (0.08%) correctly answered all six statements. The percentage of participants that answered each statement correctly varied from 8.8% for the ‘popcorn lung’ statement to 50.5% for the ‘water vapor’ statement (Table 2). Mean knowledge scores were slightly higher for UK participants (2.19, SD = 1.35) than US participants (2.05, SD = 1.31). The effect of exposure on post-exposure knowledge differed for each experimental condition (Table 3), with ‘as or more harmful’ messages showing the greatest change compared with baseline knowledge.

**Table 2.** Percentage (*n*) of correct responses for each knowledge statement at baseline by UK (*n* = 1200) and US (*n* = 1200) participants.

	All (*N* = 2400)	US (*N* = 1200)	UK (*N* = 1200)
	Pre	Pre	Pre
Toxic chemicals	49.7 (1193)	58.1 (697)	41.3 (496)
Water vapour	50.5 (1212)	54.4 (653)	46.6 (559)
Formaldehyde	22.0 (527)	27.5 (330)	16.4 (197)
Tar	36.8 (884)	27.3 (328)	46.3 (556)
Fewer toxins	44.1 (1058)	31.5 (378)	56.7 (680)
Popcorn lung	8.8 (210)	6.3 (75)	11.3 (135)

**Table 3.** Percentage of correct responses for each knowledge statement by condition at baseline (pre-exposure) and after exposure to tweets (post-exposure).

	Toxic chemicals		Water vapour		Formaldehyde		Tar		Fewer toxins		Popcorn lung	
Condition	Pre	Post	Pre	Post	Pre	Post	Pre	Post	Pre	Post	Pre	Post
As or more harmful	49.0	54.3	50.3	58.0	19.3	24.8	34.7	29.3	44.7	29.3	8.7	6.8
Completely harmless	52.5	46.7	50.2	47.0	23.2	20.8	38.2	37.8	41.3	43.0	9.2	12.1
Uncertainty	46.8	46.7	49.3	48.3	22.0	21.8	38.0	33.5	44.3	38.8	8.2	8.2
Control	50.5	43.3	52.2	49.2	23.3	22.3	36.5	36.7	46.0	44.5	9.0	9.3

Table 4 summarizes the results of the regression analyses. There was strong evidence that participants exposed to ‘as or more harmful’ messages compared to the control condition had greater odds of answering correctly post-exposure that e-cigarettes contain toxic chemicals (OR = 2.14, 95% CI: 1.60, 2.86, *p* ≤ 0.001), do not contain only water vapor (OR = 2.70, 95% CI: 1.88, 3.87, *p* ≤ 0.001) and contain formaldehyde (OR = 1.92, 95% CI: 1.29, 2.84, *p* ≤ 0.001). Conversely, these participants had reduced odds of answering correctly post-exposure that e-cigarettes do not contain tar (OR = 0.56, 95% CI: 0.39, 0.79, *p* ≤ 0.001) and contain fewer toxins than regular cigarettes toxins’ (OR = 0.33, 95%CI: 0.23, 0.45, *p* ≤ 0.001). Participants exposed to ‘completely harmless’ messages compared to the control condition had greater odds of answering correctly post-exposure that e-cigarettes do not lead to popcorn lung (OR = 1.65, 95% CI: 1.01, 2.68, *p* = 0.046). Participants exposed to ‘uncertainty’ messages had increased odds of answering correctly post-exposure that e-cigarettes contain toxic chemicals (OR = 1.43, 95% CI: 1.07, 1.91, *p* = 0.015), and reduced odds of answering correctly post-exposure that e-cigarettes do not contain tar (OR = 0.66, 95% CI: 0.47, 0.94, *p* = 0.023) and contain fewer toxins than regular cigarettes (OR = 0.70, 95% CI: 0.51, 0.96, *p* = 0.028).

**Table 4.** Adjusted logistic regression analyses predicting knowledge for each statement post-exposure by experimental condition, stratified by country and controlling for baseline measures of knowledge.

	Total sample (*n* = 2400)			US (*n* = 1200)			UK (*n* = 1200)		
	OR	95% CI	*p*	OR	95% CI	*p*	OR	95% CI	*p*
Toxic chemicals									
Control (referent)									
As or more harmful	2.144	[1.604, 2.864]	≤0.001	1.982	[1.304, 3.015]	≤0.001	2.357	[1.572, 3.533]	≤0.001
Completely harmless	1.145	[0.861, 1.523]	0.352	1.011	[0.674, 1.516]	0.959	1.305	[0.871, 1.956]	0.197
Uncertainty	1.429	[1.071, 1.905]	0.015	1.268	[0.840, 1.912]	0.258	1.615	[1.076, 2.426]	0.021
Water vapor									
Control (referent)									
As or more harmful	2.702	[1.884, 3.874]	≤0.001	1.804	[1.110, 2.930]	0.017	4.502	[2.589, 7.830]	≤0.001
Completely harmless	0.940	[0.660, 1.339]	0.732	0.826	[0.510, 1.338]	0.438	1.078	[0.640, 1.815]	0.777
Uncertainty	1.142	[0.801, 1.631]	0.463	0.878	[0.542, 1.423]	0.598	1.578	[0.922, 2.699]	0.096
Formaldehyde									
Control (referent)									
As or more harmful	1.917	[1.293, 2.842]	≤0.001	2.008	[1.188, 3.396]	0.009	1.813	[0.995, 3.304]	0.052
Completely harmless	0.839	[0.560, 1.256]	0.393	1.034	[0.608, 1.759]	0.901	0.626	[0.332, 1.182]	0.149
Uncertainty	1.061	[0.710, 1.587]	0.772	1.014	[0.596, 1.727]	0.959	1.128	[0.609, 2.092]	0.701
Tar									
Control (referent)									
As or more harmful	0.555	[0.388, 0.792]	≤0.001	0.693	[0.412, 1.168]	0.169	0.445	[0.272, 0.729]	≤0.001
Completely harmless	0.993	[0.700, 1.409]	0.970	1.051	[0.631, 1.749]	0.849	0.924	[0.567, 1.505]	0.751
Uncertainty	0.664	[0.467, 0.944]	0.023	0.682	[0.410, 1.136]	0.141	0.648	[0.397, 1.058]	0.083
Fewer toxins									
Control (referent)									
As or more harmful	0.327	[0.237, 0.452]	≤0.001	0.302	[0.186, 0.490]	≤0.001	0.355	[0.230, 0.548]	≤0.001
Completely harmless	1.151	[0.836, 1.585]	0.388	0.874	[0.554, 1.378]	0.561	1.498	[0.953, 2.356]	0.080
Uncertainty	0.698	[0.507, 0.961]	0.028	0.603	[0.379, 0.959]	0.032	0.796	[0.512, 1.240]	0.309
Popcorn lung									
Control (referent)									
As or more harmful	0.590	[0.342, 1.020]	0.059	0.623	[0.251, 1.546]	0.307	0.494	[0.240, 1.014]	0.055
Completely harmless	1.646	[1.009, 2.684]	0.046	2.370	[1.150, 4.884]	0.019	1.211	[0.617, 2.377]	0.577
Uncertainty	0.874	[0.517, 1.479]	0.617	1.399	[0.642. 3.047]	0.398	0.571	[0.276, 1.181]	0.131

After stratification by country, only UK participants exposed to ‘as or more harmful’ messages compared to the control condition had greater odds of answering correctly post-exposure that e-cigarettes do not contain only water vapor (OR = 4.50, 95% CI: 2.59, 7.83, *p* ≤ 0.001) and reduced odds of answering correctly post-exposure that e-cigarettes do not contain tar (OR = 0.45, 95% CI: 0.27, 0.73, *p* ≤ 0.001). Only US participants exposed to ‘as or more harmful’ messages compared to the control condition had greater odds of answering correctly post-exposure that e-cigarettes contain formaldehyde (OR = 2.01, 95% CI: 1.19, 3.40, *p* = 0.009).

### Perceived harm perceptions of switching completely to e-cigarettes

The percentage of people choosing ‘likely’ or ‘extremely likely’ was relatively high for each health condition: lung cancer (46.63%), heart disease (41.96%), mouth or throat cancer (42.04%), COPD (46.83%) and stroke (38.96%). Baseline harm perceptions were greatest for lung cancer (mean = 3.39, SD = 1.03) and COPD (mean = 3.39, SD = 1.04), and lowest for stroke (mean = 3.24, SD = 1.02). US participants had greater mean scores than UK participants at baseline (Table 5). There was strong evidence that exposure to ‘as or more harmful’ messages versus control messages increased harm perceptions for lung cancer (β = .31, 95% CI: 0.23, 0.40, *p* ≤ 0.001), heart disease (β = 0.17, 95% CI: 0.08, 0.25, *p* ≤ 0.001), mouth or throat cancer (β = 0.22, 95% CI: 0.14, 0.31, *p* ≤ 0.001), COPD (β = 0.19, 95% CI: 0.11, 0.28, *p* ≤ 0.001) and stroke (β = 0.19, 95% CI: 0.11, 0.28, *p* ≤ 0.001). After stratification, only UK participants showed increased harm perceptions for heart disease (β = 0.26, 95% CI: 0.15, 0.37, *p* ≤ 0.001), mouth or throat cancer (β = 0.33, 95% CI: 0.22, 0.44, *p* ≤ 0.001) and stroke (β = 0.29, 95% CI: 0.18, 0.40, *p* ≤ 0.001). There was limited evidence that exposure to ‘completely harmless’ or ‘uncertainty’ messages influenced harm perceptions (see Table 6).

**Table 5.** Mean relative harm perceptions and harm perceptions of five diseases by experimental condition and country pre- and post-exposure.

	US				UK			
Harm perceptions: Mean (SD)	As or more harmful	Completely harmless	Uncertainty	Control	As or more harmful	Completely harmless	Uncertainty	Control
Lung Cancer	(*n* = 300)	(*n* = 300)	(*n* = 300)	(*n* = 300)	(*n* = 300)	(*n* = 300)	(*n* = 300)	(*n* = 300)
Pre-exposure	3.54 (1.10)	3.64 (1.02)	3.69 (1.03)	3.56 (1.08)	3.14 (0.96)	3.16 (0.94)	3.06 (0.97)	3.30 (0.95)
Post-exposure	3.81 (1.00)	3.54 (1.01)	3.67 (1.04)	3.55 (1.02)	3.45 (1.02)	3.13 (0.98)	3.06 (1.01)	3.20 (0.99)
Heart Disease	(*n* = 300)	(*n* = 300)	(*n* = 300)	(*n* = 300)	(*n* = 300)	(*n* = 300)	(*n* = 300)	(*n* = 300)
Pre-exposure	3.51 (1.06)	3.56 (0.99)	3.58 (1.04)	3.52 (1.06)	3.01 (0.91)	3.00 (0.96)	2.97 (0.92)	3.20 (0.93)
Post-exposure	3.63 (1.07)	3.53 (1.00)	3.64 (1.02)	3.56 (0.99)	3.25 (0.99)	2.98 (0.98)	2.93 (0.95)	3.13 (0.96)
Mouth or Throat Cancer	(*n* = 300)	(*n* = 300)	(*n* = 300)	(*n* = 300)	(*n* = 300)	(*n* = 300)	(*n* = 300)	(*n* = 300)
Pre-exposure	3.44 (1.08)	3.48 (1.05)	3.52 (1.08)	3.41 (1.09)	3.08 (0.96)	3.12 (0.94)	3.00 (0.95)	3.23 (0.94)
Post-exposure	3.58 (1.04)	3.39 (1.02)	3.52 (1.04)	3.45 (1.06)	3.32 (1.01)	3.01 (0.96)	2.98 (0.98)	3.10 (0.98)
COPD	(*n* = 300)	(*n* = 300)	(*n* = 300)	(*n* = 300)	(*n* = 300)	(*n* = 300)	(*n* = 300)	(*n* = 300)
Pre-exposure	3.53 (1.08)	3.72 (1.01)	3.70 (1.04)	3.61 (1.07)	3.18 (0.97)	3.11 (0.99)	3.03 (0.96)	3.26 (0.96)
Post-exposure	3.76 (1.02)	3.60 (1.05)	3.69 (1.02)	3.64 (1.00)	3.33 (1.06)	3.09 (0.99)	3.05 (0.99)	3.17 (0.98)
Stroke	(*n* = 300)	(*n* = 300)	(*n* = 300)	(*n* = 300)	(*n* = 300)	(*n* = 300)	(*n* = 300)	(*n* = 300)
Pre-exposure	3.43 (1.08)	3.53 (1.01)	3.51 (1.06)	3.43 (1.06)	3.00 (0.89)	2.93 (0.96)	2.90 (0.91)	3.16 (0.90)
Post-exposure	3.60 (1.06)	3.45 (0.96)	3.52 (1.07)	3.48 (1.00)	3.22 (0.98)	2.94 (0.96)	2.93 (0.94)	3.06 (0.97)

**Table 6.** Adjusted regression analyses predicting post-exposure harm perceptions for each disease by experimental condition, stratified by country and controlling for baseline measures of harm perceptions.

	Total sample (*n* = 2400)			US (*n* = 1200)			UK (*n* = 1200)		
	β	95% CI	*p*	β	95% CI	*p*	β	95% CI	*p*
Lung cancer									
Control (referent)									
As or more harmful	0.313	[0.229, 0.398]	≤0.001	0.271	[0.150, 0.392]	≤0.001	0.360	[0.244, 0.476]	≤0.001
Completely harmless	−0.016	[−0.101, 0.068]	0.702	−0.056	[−0.177, 0.065]	0.366	0.031	[−0.085, 0.147]	0.597
Uncertainty	0.024	[−0.060, 0.108]	0.574	0.036	[−0.085, 0.158]	0.555	0.025	[−0.091, 0.142]	0.668
Heart disease									
Control (referent)									
As or more harmful	0.167	[ 0.084, 0.250]	≤0.001	0.080	[−0.045, 0.204]	0.209	0.261	[ 0.154, 0.368]	≤0.001
Completely harmless	−0.035	[−0.118, 0.047]	0.402	−0.063	[−0.188, 0.061]	0.320	0.005	[−0.102, 0.112]	0.930
Uncertainty	0.001	[−0.082, 0.084]	0.980	0.040	[−0.085, 0.164]	0.533	−0.022	[−0.130, 0.085]	0.681
Mouth or throat cancer									
Control (referent)									
As or more harmful	0.220	[ 0.135, 0.304]	≤0.001	0.118	[−0.009, 0.244]	0.068	0.331	[ 0.221, 0.441]	≤0.001
Completely harmless	−0.060	[−0.144, 0.024]	0.163	−0.104	[−0.231, 0.022]	0.106	−0.006	[−0.116, 0.104]	0.912
Uncertainty	0.019	[−0.066, 0.103]	0.665	−0.002	[−0.128, 0.125]	0.979	0.057	[−0.053, 0.167]	0.312
COPD									
Control (referent)									
As or more harmful	0.198	[ 0.114, 0.282]	≤0.001	0.168	[ 0.043, 0.293]	0.009	0.224	[ 0.115, 0.334]	≤0.001
Completely harmless	−0.046	[−0.130, 0.038]	0.280	−0.113	[−0.238, 0.013]	0.078	0.037	[−0.073, 0.147]	0.507
Uncertainty	0.018	[−0.066, 0.102]	0.681	−0.003	[−0.128, 0.123]	0.967	0.056	[−0.053, 0.166]	0.314
Stroke									
Control (referent)									
As or more harmful	0.197	[ 0.114, 0.279]	≤0.001	0.115	[−0.009, 0.238]	0.069	0.287	[ 0.179, 0.395]	≤0.001
Completely harmless	−0.035	[−0.117, 0.048]	0.408	−0.100	[−0.223, 0.024]	0.133	0.048	[−0.060, 0.156]	0.382
Uncertainty	0.018	[−0.064, 0.101]	0.666	−0.013	[−0.136, 0.111]	0.842	0.067	[−0.041, 0.175]	0.226

Sensitivity analyses for both knowledge and harm were also conducted owing to differences in baseline measures between US and UK participants. US participants tended to be older, more female, more educated, and more racially diverse. However, no substantial differences were found (see Appendix A and B).

## Discussion

This study demonstrated that brief exposure to misinformation on Twitter about e-cigarette harms can influence adult smokers’ knowledge of harms and harm perceptions of e-cigarette use, supporting our hypotheses. These findings contribute to a limited evidence-based exploring the effect of social media misinformation on knowledge and harm perceptions of e-cigarettes.

The finding that ‘as or more harmful’ misinformation had a greater effect on knowledge and perceptions than ‘completely harmless’ misinformation or uncertainty messages could be explained by negativity bias.^25^ This argues that negative information is more memorable and compelling than positive information and is assigned a greater weight in belief formation.^25^ An alternative theory is confirmation bias, which holds that individuals tend to accept information that reflects their preconceived beliefs over information that challenges them, even when the validity of that information is questioned by others.^26^ UK participants showed greater increases in harm perceptions after exposure to ‘as or more harmful’ misinformation. This is surprising given the more permissive e-cigarette context in the UK and may be explained by a ceiling effect, as US participants had higher baseline harm perceptions. Uncertainty messages showed relatively weak effects on both knowledge and harm perceptions. In a recent survey, 17.6% of UK adult smokers were uncertain of the relative harms of e-cigarettes.^27^ Consequently, exposure to the stimuli may have had less of an effect on participants where uncertainty is already prevalent. Furthermore, misinformation about e-cigarette may widen knowledge gaps, and therefore tobacco-related health disparities, given that cigarette smokers tend to be from traditionally marginalized backgrounds.^28^

Exposure to ‘as or more harmful’ misinformation was found to have opposing effects on knowledge statements. One possible explanation is that harm perceptions mediated the observed effects on knowledge, so participants who viewed ‘as or more harmful’ messages perceived higher levels of harm and therefore answered questions in the direction that indicates greater harm, regardless of whether that statement was true or false. This theory was consistent for the five statements where there was strong evidence of a change in knowledge. For example, for the statement ‘Vapor from e-cigarettes contain only water,’ more participants correctly answered post-exposure, whilst for the statement ‘E-cigarettes contain tar, which can cause lung cancer,’ more participants incorrectly answered post-exposure. No pattern was found between the content of the ‘as or more harmful’ message stimuli and knowledge for each statement; therefore, it is unlikely that particular phrases in the tweets had an effect.

These findings demonstrate that misinformation may be impeding attempts to reduce tobacco-related harms among current smokers. Correcting misinformation is complex, as definitions of misinformation can change as new evidence emerges.^29^ This can make developing strategies to identify and counteract e-cigarette misinformation challenging. Future research should focus on understanding the factors that make misinformation effective (i.e. message content, visual cues, period of exposure) across different online platforms, to determine the effect of source credibility on susceptibility to misinformation.^30^ There is evidence to suggest that misperceptions can be debunked when misinformation is tackled quickly and includes corrective information, thus e-cigarette misinformation should be corrected early on as it emerges.^31^ Social media platforms have begun to implement algorithms that can detect false information, yet there are inherent technical difficulties in capturing misinformation accurately through these channels and addressing detected misinformation effectively and appropriately through algorithms.. There is also a changing environment for audiences to be exposed to health misinformation in addition to social media, for example, from interpersonal communication or other forms of media including the traditional news media.^32–34^ Campaigns that focus on increasing public awareness of misinformation and educating users on trustworthy information sources may be a more effective and sustainable way to tackle misinformation.^31^

This study has certain limitations. Firstly, the study was limited to stimuli from one social media site, Twitter, and does not account for differences in how misinformation spreads across social media sites. However, this study provides insight on how similar studies can be carried out on other social media platforms. Additionally, overall knowledge was low, with 60.0% of participants answering ≤2 statements correctly at baseline. This skewed the distribution of scores and created a floor effect, indicating that the test items may have been too difficult for participants. Lastly, the skewed distribution of ethnic groups also means these findings do not accurately represent the general population and could reflect self-selection bias in enrolling into the Prodege research panel. There are important ethical implications associated with exposing participants to misinformation,^25^ and steps were taken by the research team to mitigate any harmful effects of exposure, such as providing corrective information at the end of the study survey.

In summary, this study provides evidence from an online randomized controlled experiment that exposure to e-cigarette misinformation can influence adult smokers’ knowledge and harm perceptions of using e-cigarettes. These findings show that e-cigarette misinformation has the potential to undermine public health efforts to reduce the harms associated with tobacco smoking. Correcting misperceptions can be difficult and the influence of misinformation can be long-lasting.^11^ Future research should focus on the mechanism of action of misinformation, for example: what individual's factors contribute to the change in knowledge of harms and harms perceptions that we observed, how to prevent misinformation before people are exposed to it, and effectively counteract it after the exposure. Implementing strategies to correct misinformation online can have important population-level implications not just in relation to e-cigarettes, but for a wide range of public health topics.^35^

## Sensitivity analysis

**Appendix A.** Sensitivity analyses: adjusted regression analyses predicting knowledge of e-cigarettes, including country as a covariate and controlling for baseline measures of the study outcome.

	S1: Toxic Chemicals (n = 2,400)			S2: Water Vapor (n = 2,400)			S3: Formaldehyde (n = 2,400)		
	OR	95% CI	p	OR	95% CI	p	OR	95% CI	p
Control (referent)									
As or more harmful	2.145	[1.604, 2.866]	≤0.001	2.714	[1.890, 3.896]	≤0.001	1.923	[1.296, 2.854]	≤0.001
Completely harmless	1.147	[0.862, 1.526]	0.348	0.942	[0.661, 1.342]	0.742	0.841	[0.561, 1.262]	0.403
Uncertainty	1.429	[1.071, 1.906]	0.015	1.143	[0.800, 1.633]	0.464	1.062	[0.709, 1.591]	0.770
	**S4: Tar (n = 2,400)**			**S5: Fewer Toxins (n = 2,400)**			**S6: Popcorn Lung (n = 2,400)**		
	**OR**	**95% CI**	**p**	**OR**	**95% CI**	**p**	**OR**	**95% CI**	**p**
Control (referent)									
As or more harmful	0.547	[0.383, 0.783]	≤0.001	0.326	[0.236, 0.451]	≤0.001	0.580	[0.335, 1.005]	0.052
Completely harmless	0.989	[0.696, 1.404]	0.950	1.148	[0.834, 1.581]	0.397	1.649	[1.012, 2.688]	0.045
Uncertainty	0.666	[0.468, 0.947]	0.024	0.696	[0.505, 0.959]	0.027	0.871	[0.515, 1.474]	0.608

**Appendix B.** Sensitivity analyses: adjusted regression analyses predicting relative harm of e-cigarettes and harm perceptions of five diseases, including country as a covariate and controlling for baseline measures of the respective outcome.

	Lung Cancer (n = 2,400)			Heart Disease (n = 2,400)				
	β	95% CI	p	β	95% CI	p		
Control (referent)								
As or more harmful	0.312	[ 0.228, 0.396]	≤0.001	0.165	[ 0.082, 0.247]	≤0.001		
Completely harmless	−0.017	[-0.101, 0.067]	0.694	−0.037	[-0.119, 0.045]	0.378		
Uncertainty	0.023	[0.060, 0.107]	0.584	−0.001	[-0.083, 0.082]	0.987		
**Mouth or Throat Cancer (n = 2,400)**			**COPD (n = 2,400)**			**Stroke (n = 2,400)**		
**β**	**95% CI**	**p**	**β**	**95% CI**	**p**	**β**	**95% CI**	**p**
0.219	[0.135, 0.303]	≤0.001	0.196	[ 0.112, 0.279]	≤0.001	0.195	[ 0.113, 0.277]	≤0.001
−0.060	[-0.144, 0.024]	0.159	−0.047	[-0.130, 0.037]	0.273	−0.036	[-0.118, 0.046]	0.392
0.018	[-0.066, 0.102]	0.675	0.016	[-0.067, 0.100]	0.703	0.017	[-0.065, 0.099]	0.692

## References

[1] Glantz SA Bareham DW . E-cigarettes: Use, effects on smoking, risks, and policy implications. Annu Rev Public Health 2018; 39: 215–235. 29323609 10.1146/annurev-publhealth-040617-013757 PMC6251310

[2] Mcneill A Brose LS Calder R , et al. Vaping in England, an Evidence Update, February 2019. A Report Commissioned by Public Health England. London; 2019.

[3] Erku DA Kisely S Morphett K , et al. Framing and scientific uncertainty in nicotine vaping product regulation: An examination of competing narratives among health and medical organisations in the UK, Australia and New Zealand. Int J Drug Policy 2020; 78: 102699. 32086156 10.1016/j.drugpo.2020.102699

[4] Public Health England. Vaping in England: Evidence update February 2021. https://www.gov.uk/government/publications/vaping-in-england-evidence-update-february-2021. Published 2021.

[5] Cornelius ME Wang TW Jamal A , et al. Tobacco product use among adults – United States, 2019. MMWR Morb Mortal Wkly Rep 2020; 69: 1736–1742. 33211681 10.15585/mmwr.mm6946a4 PMC7676638

[6] Abrams DB Glasser AM Pearson JL , et al. Harm minimization and tobacco control: Reframing societal views of nicotine use to rapidly save lives. Annu Rev Public Health 2018; 39: 193–213. 29323611 10.1146/annurev-publhealth-040617-013849 PMC6942997

[7] Huang J Feng B Weaver SR , et al. Changing perceptions of harm of e-cigarette vs cigarette use among adults in 2 US national surveys from 2012 to 2017. JAMA Netw Open 2019; 2: e191047–e191047. 30924893 10.1001/jamanetworkopen.2019.1047 PMC6450305

[8] Nyman AL Huang J Weaver SR , et al. Perceived comparative harm of cigarettes and electronic nicotine delivery systems. JAMA Netw Open 2019; 2: e1915680. 31747029 10.1001/jamanetworkopen.2019.15680 PMC6902805

[9] Perski O Beard E Brown J . Association between changes in harm perceptions and e-cigarette use among current tobacco smokers in England: A time series analysis. BMC Med 2020; 18: 98. 32370755 10.1186/s12916-020-01565-2 PMC7201665

[10] Shin J Jian L Driscoll K , et al. The diffusion of misinformation on social media. Comput Hum Behav 2018; 83: 278–287.

[11] Wang Y McKee M Torbica A , et al. Systematic literature review on the spread of health-related misinformation on social media. Soc Sci Med 2019; 240. doi:10.1016/j.socscimed.2019.112552 PMC7117034 31561111

[12] Soroush V Deb R Sinan A . The spread of true and false news online. Science 2018; 359: 1146–1151. 29590045 10.1126/science.aap9559

[13] Pepper JK Squiers LB Peinado SC , et al. Impact of messages about scientific uncertainty on risk perceptions and intentions to use electronic vaping products. Addict Behav 2019; 91: 136–140. 30389200 10.1016/j.addbeh.2018.10.025

[14] Wright C Williams P Elizarova O , et al. Effects of brief exposure to misinformation about e-cigarette harms on twitter: A randomised controlled experiment. BMJ Open 2021; 11: 1–14. doi:10.1136/bmjopen-2020-045445 PMC8413940 34470790

[15] Liu J Wright C Williams P , et al. Smokers’ likelihood to engage with information and misinformation on twitter about the relative harms of e-cigarette use: Results from a randomized controlled trial. JMIR Public Heal Surveill 2021; 7: 1–10. 10.2196/27183 PMC8734921 34931999

[16] Liu J Wright C Elizarova O , et al. Emotional responses and perceived relative harm mediate the effect of exposure to misinformation about e-cigarettes on twitter and intention to purchase e-cigarettes among adult smokers. Int J Environ Res Public Health 2021; 18: 1–13. 10.3390/ijerph182312347 PMC8656833 34886071

[17] Sanders-Jackson AN Tan ASL Bigman CA , et al. Knowledge about e-cigarette constituents and regulation: Results from a national survey of U.S. young adults. Nicotine Tob Res 2015; 17: 1247–1254. 25542915 10.1093/ntr/ntu276 PMC4592338

[18] Rohde JA Noar SM Horvitz C , et al. The role of knowledge and risk beliefs in adolescent e-cigarette use: A pilot study. Int J Environ Res Public Health 2018; 15: 30. 10.3390/ijerph15040830 PMC5923872 29690606

[19] Webb Hooper M Kolar SK . Racial/ethnic differences in electronic cigarette use and reasons for use among current and former smokers: Findings from a community-based sample. Int J Environ Res Public Health 2016; 13: 1009. 10.3390/ijerph13101009 PMC5086748 27754449

[20] Franks AM Hawes WA McCain KR , et al. Electronic cigarette use, knowledge, and perceptions among health professional students. Curr Pharm Teach Learn 2017; 9: 1003–1009. 29233367 10.1016/j.cptl.2017.07.023

[21] Lin W Martinez SA Ding K , et al. Knowledge and perceptions of tobacco-related harm associated with intention to quit among cigarette smokers, e-cigarette users, and dual users: Findings from the US population assessment of tobacco and health (PATH) wave 1. Subst Use Misuse 2021; 56: 464–470. 33594931 10.1080/10826084.2021.1879145

[22] Pepper JK Emery SL Ribisl KM , et al. How risky is it to use e-cigarettes? Smokers’ beliefs about their health risks from using novel and traditional tobacco products. J Behav Med 2015; 38: 318–326. 25348584 10.1007/s10865-014-9605-2 PMC4520302

[23] Erdfelder E Faul F Buchner A . GPOWER: A general power analysis program. Behav Res Methods, Instruments, Comput 1996; 28: 1–11.

[24] R Team Core. R: A language and environment for statistical computing. 2020.

[25] Walter N Tukachinsky R . A meta-analytic examination of the continued influence of misinformation in the face of correction: How powerful is it, why does it happen, and how to stop it? Communic Res 2019; 47: 155–177.

[26] Karduni A Wesslen R Santhanam S , et al. Can you verifi this? Studying uncertainty and decision-making about misinformation using visual analytics. Proc Int AAAI Conf Web Soc Media 2018; 12. https://ojs.aaai.org/index.php/ICWSM/article/view/15014 (accessed 24 March, 2022)

[27] Wilson S Partos T McNeill A , et al. Harm perceptions of e-cigarettes and other nicotine products in a UK sample. Addiction 2019; 114: 879–888. 30609154 10.1111/add.14502 PMC6491935

[28] Tan ASL Bigman CA . Misinformation about commercial tobacco products on social media—implications and research opportunities for reducing tobacco-related health disparities. Am J Public Health 2020; 110: S281–S283. 33001728 10.2105/AJPH.2020.305910 PMC7532322

[29] Swire-Thompson B Lazer D . Public health and online misinformation: Challenges and recommendations. Annu Rev Public Health 2020; 41: 433–451. 31874069 10.1146/annurev-publhealth-040119-094127

[30] Bode L Vraga EK . See something, say something: Correction of global health misinformation on social media. Health Commun 2018; 33: 1131–1140. 28622038 10.1080/10410236.2017.1331312

[31] van der Meer TGLA Jin Y . Seeking formula for misinformation treatment in public health crises: The effects of corrective information type and source. Health Commun 2020; 35: 560–575. 30761917 10.1080/10410236.2019.1573295

[32] Park S Fisher C Lee JY , et al. COVID-19: Australian news and misinformation. 2020. https://doi.org/APO-306728

[33] Wu Y Kuru O Campbell SW , et al. Explaining health misinformation belief through news, social, and alternative health media use: The moderating roles of need for cognition and faith in intuition. Health Commun January 2022: 1–14. doi:10.1080/10410236.2021.2010891 34978236

[34] Bryanov K Vziatysheva V . Determinants of individuals’ belief in fake news: A scoping review determinants of belief in fake news. PLoS One 2021; 16: e0253717–e0253717. 34166478 10.1371/journal.pone.0253717 PMC8224890

[35] Michela DV Alessandro B Fabiana Z , et al. The spreading of misinformation online. Proc Natl Acad Sci 2016; 113: 554–559. 26729863 10.1073/pnas.1517441113 PMC4725489

